# Supplementary material for: Heparin-based hydrogel scaffolding alters the transcriptomic profile and increases the chemoresistance of MDA-MB-231 triple-negative breast cancer cells
Source: Biomater Sci. 2020 Feb 13;8(10):2786–96. doi: 10.1039/c9bm01481k (PMC7497406; doi:10.1039/c9bm01481k)
Supplement: Supplementary file 2 [file BM-008-C9BM01481K-s002.zip › Supplementary File 4/HP-BhydrogelvControl/Pathways/my_analysis.Gsea.1545201079907/HALLMARK_DNA_REPAIR.html]

Details for gene set HALLMARK\_DNA\_REPAIR[GSEA]

|  || Dataset | expr.class.cls#HYD\_versus\_CONTROL.class.cls#HYD\_versus\_CONTROL\_repos |
| Phenotype | class.cls#HYD\_versus\_CONTROL\_repos |
| Upregulated in class | CONTROL |
| GeneSet | HALLMARK\_DNA\_REPAIR |
| Enrichment Score (ES) | -0.33165753 |
| Normalized Enrichment Score (NES) | -1.5192845 |
| Nominal p-value | 0.0 |
| FDR q-value | 0.01884876 |
| FWER p-Value | 0.108 |
Table: GSEA Results Summary

  

Fig 1: Enrichment plot: HALLMARK\_DNA\_REPAIR      
 Profile of the Running ES Score & Positions of GeneSet Members on the Rank Ordered List

  

| PROBE | DESCRIPTION (from dataset) | GENE SYMBOL | GENE\_TITLE | RANK IN GENE LIST | RANK METRIC SCORE | RUNNING ES | CORE ENRICHMENT || 1 | SNAPC4 | na |  |  | 596 | 2.222 | -0.0104 | No |
| 2 | ELL | na |  |  | 631 | 2.175 | 0.0083 | No |
| 3 | RAD52 | na |  |  | 898 | 1.952 | 0.0127 | No |
| 4 | DGCR8 | na |  |  | 925 | 1.929 | 0.0294 | No |
| 5 | ZNF707 | na |  |  | 966 | 1.897 | 0.0452 | No |
| 6 | VPS37D | na |  |  | 1724 | 1.480 | 0.0193 | No |
| 7 | ADA | na |  |  | 1938 | 1.392 | 0.0212 | No |
| 8 | ADCY6 | na |  |  | 2160 | 1.292 | 0.0218 | No |
| 9 | STX3 | na |  |  | 2260 | 1.254 | 0.0284 | No |
| 10 | POM121 | na |  |  | 2397 | 1.209 | 0.0326 | No |
| 11 | TAF1C | na |  |  | 2484 | 1.179 | 0.0392 | No |
| 12 | XPC | na |  |  | 2677 | 1.124 | 0.0397 | No |
| 13 | POLR2A | na |  |  | 2700 | 1.114 | 0.0490 | No |
| 14 | SUPT5H | na |  |  | 3031 | 1.029 | 0.0413 | No |
| 15 | VPS28 | na |  |  | 3037 | 1.027 | 0.0507 | No |
| 16 | TAF6 | na |  |  | 3139 | 1.002 | 0.0548 | No |
| 17 | GUK1 | na |  |  | 3287 | 0.967 | 0.0562 | No |
| 18 | POLR2E | na |  |  | 3333 | 0.958 | 0.0629 | No |
| 19 | NPR2 | na |  |  | 3454 | 0.927 | 0.0653 | No |
| 20 | ERCC3 | na |  |  | 3600 | 0.894 | 0.0661 | No |
| 21 | ERCC2 | na |  |  | 3628 | 0.886 | 0.0730 | No |
| 22 | DDB2 | na |  |  | 3676 | 0.875 | 0.0787 | No |
| 23 | POLD1 | na |  |  | 3872 | 0.839 | 0.0764 | No |
| 24 | LIG1 | na |  |  | 3913 | 0.830 | 0.0821 | No |
| 25 | POLA2 | na |  |  | 4005 | 0.811 | 0.0849 | No |
| 26 | GTF3C5 | na |  |  | 4371 | 0.738 | 0.0727 | No |
| 27 | SURF1 | na |  |  | 4879 | 0.645 | 0.0522 | No |
| 28 | AK1 | na |  |  | 4927 | 0.638 | 0.0557 | No |
| 29 | CCNO | na |  |  | 5102 | 0.613 | 0.0523 | No |
| 30 | NFX1 | na |  |  | 5208 | 0.598 | 0.0524 | No |
| 31 | POLL | na |  |  | 5369 | 0.574 | 0.0494 | No |
| 32 | SNAPC5 | na |  |  | 5540 | 0.546 | 0.0456 | No |
| 33 | ADRM1 | na |  |  | 5890 | 0.501 | 0.0320 | No |
| 34 | SAC3D1 | na |  |  | 6253 | 0.451 | 0.0173 | No |
| 35 | TARBP2 | na |  |  | 6509 | 0.415 | 0.0078 | No |
| 36 | RAE1 | na |  |  | 6569 | 0.407 | 0.0085 | No |
| 37 | GTF2F1 | na |  |  | 6759 | 0.385 | 0.0022 | No |
| 38 | NME3 | na |  |  | 7128 | 0.344 | -0.0138 | No |
| 39 | ZWINT | na |  |  | 7332 | 0.322 | -0.0215 | No |
| 40 | ITPA | na |  |  | 7633 | 0.287 | -0.0345 | No |
| 41 | POLH | na |  |  | 7767 | 0.278 | -0.0389 | No |
| 42 | POLR2H | na |  |  | 7921 | 0.259 | -0.0445 | No |
| 43 | PRIM1 | na |  |  | 7968 | 0.252 | -0.0445 | No |
| 44 | REV3L | na |  |  | 8568 | 0.197 | -0.0741 | No |
| 45 | POLD4 | na |  |  | 8664 | 0.184 | -0.0773 | No |
| 46 | NME4 | na |  |  | 9008 | 0.153 | -0.0939 | No |
| 47 | CANT1 | na |  |  | 9012 | 0.153 | -0.0926 | No |
| 48 | TK2 | na |  |  | 9052 | 0.147 | -0.0933 | No |
| 49 | POLR2D | na |  |  | 9591 | 0.091 | -0.1207 | No |
| 50 | RAD51 | na |  |  | 9612 | 0.087 | -0.1209 | No |
| 51 | TYMS | na |  |  | 9817 | 0.062 | -0.1310 | No |
| 52 | RFC5 | na |  |  | 9822 | 0.061 | -0.1307 | No |
| 53 | BCAM | na |  |  | 9832 | 0.060 | -0.1306 | No |
| 54 | ERCC5 | na |  |  | 9859 | 0.057 | -0.1314 | No |
| 55 | POLE4 | na |  |  | 9962 | 0.044 | -0.1363 | No |
| 56 | TAF13 | na |  |  | 10107 | 0.028 | -0.1436 | No |
| 57 | POLA1 | na |  |  | 10108 | 0.027 | -0.1434 | No |
| 58 | CMPK2 | na |  |  | 10261 | 0.015 | -0.1512 | No |
| 59 | NT5C | na |  |  | 10664 | -0.025 | -0.1721 | No |
| 60 | EDF1 | na |  |  | 10719 | -0.031 | -0.1746 | No |
| 61 | FEN1 | na |  |  | 10780 | -0.035 | -0.1774 | No |
| 62 | RFC2 | na |  |  | 10894 | -0.053 | -0.1828 | No |
| 63 | GMPR2 | na |  |  | 11050 | -0.072 | -0.1903 | No |
| 64 | POLD3 | na |  |  | 11139 | -0.085 | -0.1941 | No |
| 65 | POLR1C | na |  |  | 11356 | -0.097 | -0.2045 | No |
| 66 | APRT | na |  |  | 11391 | -0.100 | -0.2054 | No |
| 67 | TAF10 | na |  |  | 11507 | -0.112 | -0.2104 | No |
| 68 | NME1 | na |  |  | 11542 | -0.117 | -0.2111 | No |
| 69 | USP11 | na |  |  | 11584 | -0.124 | -0.2120 | No |
| 70 | VPS37B | na |  |  | 11608 | -0.127 | -0.2121 | No |
| 71 | UPF3B | na |  |  | 11682 | -0.136 | -0.2146 | No |
| 72 | TAF12 | na |  |  | 11890 | -0.162 | -0.2240 | No |
| 73 | POLR3C | na |  |  | 12125 | -0.190 | -0.2344 | No |
| 74 | BOLA2 | na |  |  | 12374 | -0.222 | -0.2454 | No |
| 75 | SRSF6 | na |  |  | 12773 | -0.255 | -0.2639 | No |
| 76 | UMPS | na |  |  | 12776 | -0.255 | -0.2616 | No |
| 77 | BCAP31 | na |  |  | 12908 | -0.272 | -0.2659 | No |
| 78 | CLP1 | na |  |  | 12945 | -0.277 | -0.2652 | No |
| 79 | MPG | na |  |  | 12981 | -0.281 | -0.2644 | No |
| 80 | RFC3 | na |  |  | 13005 | -0.284 | -0.2629 | No |
| 81 | SSRP1 | na |  |  | 13070 | -0.294 | -0.2635 | No |
| 82 | ZNRD1 | na |  |  | 13151 | -0.307 | -0.2648 | No |
| 83 | POLR2G | na |  |  | 13421 | -0.341 | -0.2757 | No |
| 84 | RBX1 | na |  |  | 13530 | -0.357 | -0.2780 | No |
| 85 | SEC61A1 | na |  |  | 13535 | -0.358 | -0.2749 | No |
| 86 | GTF2H1 | na |  |  | 13546 | -0.359 | -0.2720 | No |
| 87 | SF3A3 | na |  |  | 14111 | -0.430 | -0.2976 | No |
| 88 | RFC4 | na |  |  | 14127 | -0.430 | -0.2943 | No |
| 89 | RALA | na |  |  | 14157 | -0.435 | -0.2917 | No |
| 90 | GPX4 | na |  |  | 14284 | -0.460 | -0.2940 | No |
| 91 | GTF2A2 | na |  |  | 14494 | -0.489 | -0.3004 | No |
| 92 | POLR2J | na |  |  | 14568 | -0.501 | -0.2995 | No |
| 93 | AAAS | na |  |  | 14672 | -0.519 | -0.3000 | No |
| 94 | SMAD5 | na |  |  | 14734 | -0.528 | -0.2983 | No |
| 95 | DDB1 | na |  |  | 15337 | -0.624 | -0.3240 | No |
| 96 | CDA | na |  |  | 15484 | -0.647 | -0.3256 | Yes |
| 97 | PDE4B | na |  |  | 15522 | -0.656 | -0.3214 | Yes |
| 98 | POLR2K | na |  |  | 15545 | -0.661 | -0.3163 | Yes |
| 99 | DGUOK | na |  |  | 15592 | -0.666 | -0.3125 | Yes |
| 100 | AK3 | na |  |  | 15863 | -0.717 | -0.3199 | Yes |
| 101 | NUDT21 | na |  |  | 15890 | -0.721 | -0.3145 | Yes |
| 102 | PCNA | na |  |  | 15928 | -0.728 | -0.3096 | Yes |
| 103 | POLR3GL | na |  |  | 15935 | -0.730 | -0.3030 | Yes |
| 104 | NUDT9 | na |  |  | 16157 | -0.780 | -0.3073 | Yes |
| 105 | RPA3 | na |  |  | 16182 | -0.784 | -0.3012 | Yes |
| 106 | PNP | na |  |  | 16242 | -0.798 | -0.2968 | Yes |
| 107 | ERCC1 | na |  |  | 16348 | -0.820 | -0.2946 | Yes |
| 108 | GTF2H3 | na |  |  | 16351 | -0.823 | -0.2870 | Yes |
| 109 | IMPDH2 | na |  |  | 16371 | -0.826 | -0.2802 | Yes |
| 110 | NCBP2 | na |  |  | 16490 | -0.851 | -0.2784 | Yes |
| 111 | ARL6IP1 | na |  |  | 16502 | -0.855 | -0.2709 | Yes |
| 112 | HCLS1 | na |  |  | 16552 | -0.866 | -0.2654 | Yes |
| 113 | MRPL40 | na |  |  | 16588 | -0.869 | -0.2590 | Yes |
| 114 | POLR1D | na |  |  | 16656 | -0.887 | -0.2542 | Yes |
| 115 | TP53 | na |  |  | 16860 | -0.944 | -0.2560 | Yes |
| 116 | TAF9 | na |  |  | 17174 | -1.039 | -0.2626 | Yes |
| 117 | POLR2C | na |  |  | 17326 | -1.093 | -0.2603 | Yes |
| 118 | RNMT | na |  |  | 17332 | -1.096 | -0.2502 | Yes |
| 119 | GTF2H5 | na |  |  | 17333 | -1.097 | -0.2399 | Yes |
| 120 | BRF2 | na |  |  | 17497 | -1.152 | -0.2377 | Yes |
| 121 | DUT | na |  |  | 17602 | -1.198 | -0.2319 | Yes |
| 122 | POLB | na |  |  | 17649 | -1.221 | -0.2228 | Yes |
| 123 | RRM2B | na |  |  | 17979 | -1.367 | -0.2272 | Yes |
| 124 | RPA2 | na |  |  | 18162 | -1.475 | -0.2229 | Yes |
| 125 | GTF2B | na |  |  | 18268 | -1.540 | -0.2139 | Yes |
| 126 | DCTN4 | na |  |  | 18275 | -1.548 | -0.1997 | Yes |
| 127 | POLR2I | na |  |  | 18515 | -1.718 | -0.1961 | Yes |
| 128 | ERCC4 | na |  |  | 18633 | -1.825 | -0.1851 | Yes |
| 129 | ERCC8 | na |  |  | 18672 | -1.868 | -0.1695 | Yes |
| 130 | TMED2 | na |  |  | 18685 | -1.879 | -0.1525 | Yes |
| 131 | SDCBP | na |  |  | 18755 | -1.959 | -0.1377 | Yes |
| 132 | EIF1B | na |  |  | 18770 | -1.974 | -0.1199 | Yes |
| 133 | CSTF3 | na |  |  | 18796 | -2.010 | -0.1023 | Yes |
| 134 | COX17 | na |  |  | 18860 | -2.099 | -0.0859 | Yes |
| 135 | TSG101 | na |  |  | 18862 | -2.104 | -0.0662 | Yes |
| 136 | CETN2 | na |  |  | 18875 | -2.131 | -0.0468 | Yes |
| 137 | HPRT1 | na |  |  | 18898 | -2.164 | -0.0276 | Yes |
| 138 | DAD1 | na |  |  | 18951 | -2.264 | -0.0091 | Yes |
| 139 | SUPT4H1 | na |  |  | 18999 | -2.338 | 0.0104 | Yes |
Table: GSEA details [plain text format]

  

Fig 2: HALLMARK\_DNA\_REPAIR      
 Blue-Pink O' Gram in the Space of the Analyzed GeneSet

  

Fig 3: HALLMARK\_DNA\_REPAIR: Random ES distribution      
 Gene set null distribution of ES for **HALLMARK\_DNA\_REPAIR**
